# Alarm Signal S100-Related Signature Is Correlated with Tumor Microenvironment and Predicts Prognosis in Glioma

**DOI:** 10.1155/2022/4968555

**Published:** 2022-05-10

**Authors:** Lin-jian Wang, Peipei Lv, Yongli Lou

**Affiliations:** ^1^Advanced Medical Research Center, Zhengzhou Central Hospital Affiliated to Zhengzhou University, Zhengzhou 450007, China; ^2^Department of Neurosurgery, Zhengzhou Central Hospital Affiliated to Zhengzhou University, Zhengzhou 450007, China; ^3^Department of Radiology, Zhengzhou Central Hospital Affiliated to Zhengzhou University, Zhengzhou 450007, China

## Abstract

Glioma are the most common malignant central nervous system tumor and are characterized by uncontrolled proliferation and resistance to therapy. Dysregulation of S100 proteins may augment tumor initiation, proliferation, and metastasis by modulating immune response. However, the comprehensive function and prognostic value of S100 proteins in glioma remain unclear. Here, we explored the expression profiles of 17 S100 family genes and constructed a high-efficient prediction model for glioma based on CGGA and TCGA datasets. Immune landscape analysis displayed that the distribution of immune scores, ESTIMATE scores, and stromal scores, as well as infiltrating immune cells (macrophages M0/M1/M2, T cell CD4+ naïve, Tregs, monocyte, neutrophil, and NK activated), were significant different between risk-score subgroups. Overall, we demonstrated the value of S100 protein-related signature in the prediction of glioma patients' prognosis and determined its relationship with the tumor microenvironment (TME) in glioma.

## 1. Introduction

Glioma are the most common primary brain tumors, accounting for about 70% of primary intracranial tumors. Grade II/III gliomas are mainly astrocytomas and oligodendrogliomas derived from astrocytes and oligodendrocytes and are defined as lower-grade gliomas (LGG) according to their malignancies. Grade IV gliomas (glioblastoma, GBM) are highly malignant and usually recur within one year after resection, and patients usually survive no more than 15 months after diagnosis [1–3]. The tumor microenvironment (TME), composed of a variety of different numbers of nontumor cells, such as mesenchymal cells, endothelial cells, stromal cells, and most importantly immune cells, plays an extremely important role in progression, recurrence, and treatment resistance [4–6]. Uncovering the key molecular mechanisms of the complex and unique microenvironment will contribute to the development of new therapeutics for glioma patients.

The S100 protein (S100s) family is composed of 25 calcium (Ca ^2+^)-binding protein members with high structural and sequence similarity. All S100s can be divided into three subgroups according to their functions, which mainly play an intracellular regulatory role, only play an extracellular regulatory role, and have both extracellular and intracellular roles [7]. S100s are involved in a variety of cellular processes such as cell proliferation, cell migration, apoptosis, inflammatory response, and calcium homeostasis [8–10] and are related to various human immune diseases such as rheumatoid arthritis and pathogenic infectious [11]. For example, S100A8 and S100A9 were found to be associated with pathogen-related tissue damage and severe cytokine storm in patients with COVID-19 [12]. In addition, a number of studies have revealed that several S100s can promote tumor progression by regulating tumor immune response [13, 14]. Of note, uncontrolled activities of some members of S100 proteins have been detected in gliomas [15–17], but it is unclear whether S100 proteins are involved in shaping the tumor microenvironment to promote glioma tumorigenesis and progression.

Here, we explored the expression profiles of 17 S100 genes in glioma and found that twelve genes (S100A1-6, S100A8-11, S100A13, S100A16, and S100Z) were aberrantly expressed in GBM relative to LGG both in the CGGA and TCGA datasets. Through univariate Cox regression analysis, the S100 family genes closely related to overall survival (OS) were identified in glioma. Significantly positive genes (*P* < 0.001) were extracted for analysis by the least absolute shrinkage and selection operator (LASSO) multivariate Cox regression algorithm. Finally, eight genes (S100A2-4, S100A8, S100A10, S100A11, S100A16, and S100Z) were screened out to establish an efficient prognostic model. According to the median risk score, patients were distributed into the low- and high-risk subgroups. Immune landscape analysis indicated that immune scores, ESTIMATE scores, and stromal scores, as well as the infiltrating immune cells (macrophages M0/M1/M2, T cell CD4+ naïve, Tregs, monocyte, neutrophil, and NK activated), were significantly different between the high- and low-risk subgroups. Moreover, the risk score and its related prognostic S100s were distinctly correlated with the immunophenotype in glioma. In summary, we identified the relationship between S100 family genes and tumor microenvironment and demonstrated the value of S100-related signature in predicting glioma prognosis.

## 2. Methods and Materials

### 2.1. Datasets

**Figure 1:**
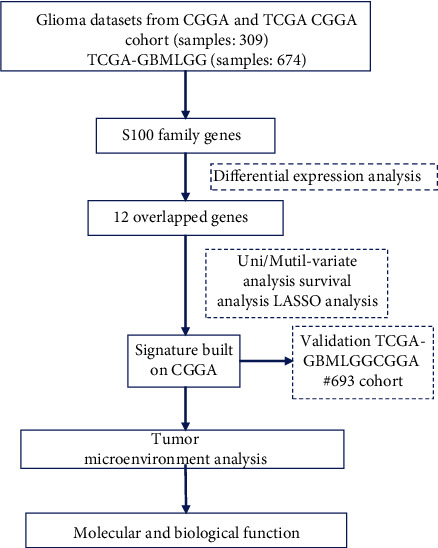
Flow chart of this study.

The clinical data and RNA-seq data of the Chinese Glioma Genome Atlas (CGGA) were obtained from the CGGA data portal (http://www.cgga.org.cn/) [18]. The merged GBMLGG data of The Cancer Genome Atlas (TCGA) was obtained from the University of California Santa Cruz (UCSC) Xena Browser (https://xenabrowser.net/datapages/) [19]. In addition, the data of GSE59612 were obtained from the GEO database (https://www.ncbi.nlm.nih.gov/geo/) [20]. All datasets were analyzed according to the flowchart (Figure 1).

### 2.2. Construction of the Risk Model

The S100 family genes that were significantly correlated with prognosis were confirmed by performing univariate cox analysis. After that, eight S100 family genes were screened out using the “glmnet” R package through LASSO method. The risk signature was constructed based on the results of LASSO, and the risk scores were calculated as follows:

(1) $$\begin{array}{c} Risk\;score=\overset{n}{\underset{i=1}{\sum }}\left({Coef}_{i}*{Exp}_{i}\right). \end{array}$$

### 2.3. Immune Landscape Analysis

The tumor microenvironment (immune, ESTIMATE, and stromal scores) of gliomas were analyzed by the “estimate” package in R software. The abundance of tumor-infiltrating immune cells were evaluated on the TIMER2 platform (http://timer.cistrome.org/) [21].

### 2.4. Transfection with siRNA

The siRNAs were synthesized by the Shanghai GenePharma Co. (Supplementary Table 1). LN229 cells were transfected with negative control siRNA and S100A4 siRNA according to the manufacturer's instructions of Lipofectamine RNAiMAX (Invitrogen). 48 hours after transfection, LN229 cells were harvested for subsequent qRT-PCR and western blot analysis, respectively.

### 2.5. RNA Extraction and qRT-PCR

Total RNA was extracted using TRIzol reagent (Invitrogen) according to the manufacturer's instructions. cDNA was reverse transcribed from total RNA using the NovoScript® Plus All-in-one 1st Strand cDNA Synthesis SuperMix (gDNA Purge) kit (Novoprotein Scientific Inc.). qRT-PCR was performed on the QuantStudio™ 6 Pro Real-Time PCR System (Applied Biosystems) using the NovoStart® SYBR qPCR SuperMix Plus kit (Novoprotein Scientific Inc.). Relative gene expression was evaluated using the 2^−*ΔΔ*CT^ method.

### 2.6. Western Blot

Cells were lysed with RIPA lysis buffer supplemented with protease inhibitors. After quantification using BCA kit, protein samples were separated by SDS-PAGE and transferred to PVDF membranes (Merck Millipore) The membranes were blocking in 5% skimmed milk for 1 hour and then incubated with anti-S100A4 (ab124805; Abcam) or anti-*α*-Tubulin (1224-1-AP; Proteintech) overnight at 4°C. After washing, the membranes were incubated with HRP-conjugated AffiniPure Goat Anti-Rabbit IgG (SA00001-2; Proteintech). Finally, the labeled proteins were detected using ECL reagent.

### 2.7. Cell Proliferation and Migration

Cell proliferation was analyzed by BeyoClick™ EdU Cell Proliferation Kit with Alexa Fluor 594. Briefly, cells were seed into glass bottom dishes and incubated with EdU for two hours at 37°C. After that, cells were incubated in Click Additive Solution for 30 minutes at room temperature in the dark. Nuclei were then stained with Hoechst 33342. Fluorescence was subsequently detected using confocal laser microscopy.

For migration assays, cells were seed into 6-well plates. After the cells were confluent, scratch the plate using pipette tips. Then, the plates were washed with PBS and incubated with serum free medium. The original images and migrated images were obtained using the inversion microscope system. The migrated area was analyzed by Image J software.

### 2.8. Statistical Analysis

The differences in infiltrating immune cells and gene expression of gliomas in different risk subgroups were analyzed by one-way ANOVA and Wilcoxon test. All statistical analyses were performed using SPSS, R, and GraphPad, and *P* < 0.05 was considered statistically significant.

## 3. Results

### 3.1. Expression Profiles of S100s in Glioma

**Figure 2:**
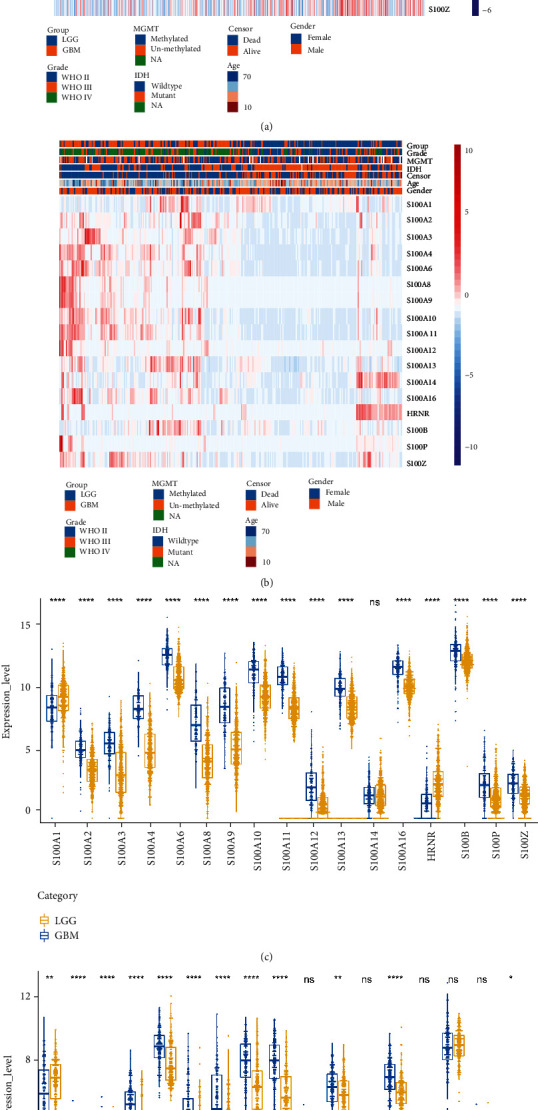
The expression profiles of 17 S100 family genes in glioma. Heatmap depicting the expression profiles of 17 S100 family genes in the (a) TCGA cohort and (b) CGGA cohort. The differential expression of 17 S100 protein family genes between LGG and GBM was explored in the (c) TCGA cohort and (d) CGGA cohort, respectively. ∗*P* < 0.05; ∗∗*P* < 0.01; ∗∗∗∗*P* < 0.0001.

S100 protein family consists of 25 calcium (Ca ^2+^)-binding protein members. Among them, 17 genes were detected to be expressed in glioma (Figure 2). We analyzed the RNA-seq data obtained from the CGGA and TCGA datasets to characterize the expression pattern of 17 S100 family genes in glioma. In the TCGA dataset, except for S100A14, all genes were differentially expressed between GBM and LGG. Compared with LGG, the expression levels of S100A2-4, S100A6, S100A8-13, S100A16, S100B, S100P, and S100Z were distinctly upregulated in GBM; meanwhile, the expression levels of S100A1 and S100A18 (HRNR) were significantly downregulated in GBM (Figures 2(a) and 2(c)). In the CGGA dataset, except that S100A12, S100A18, S100B, and S100Z were not differentially expressed between GBM and LGG, the expression of other genes was similar to that of TCGA dataset (Figures 2(b) and 2(d)).

### 3.2. Construction of Risk-Score Model

**Figure 3:**
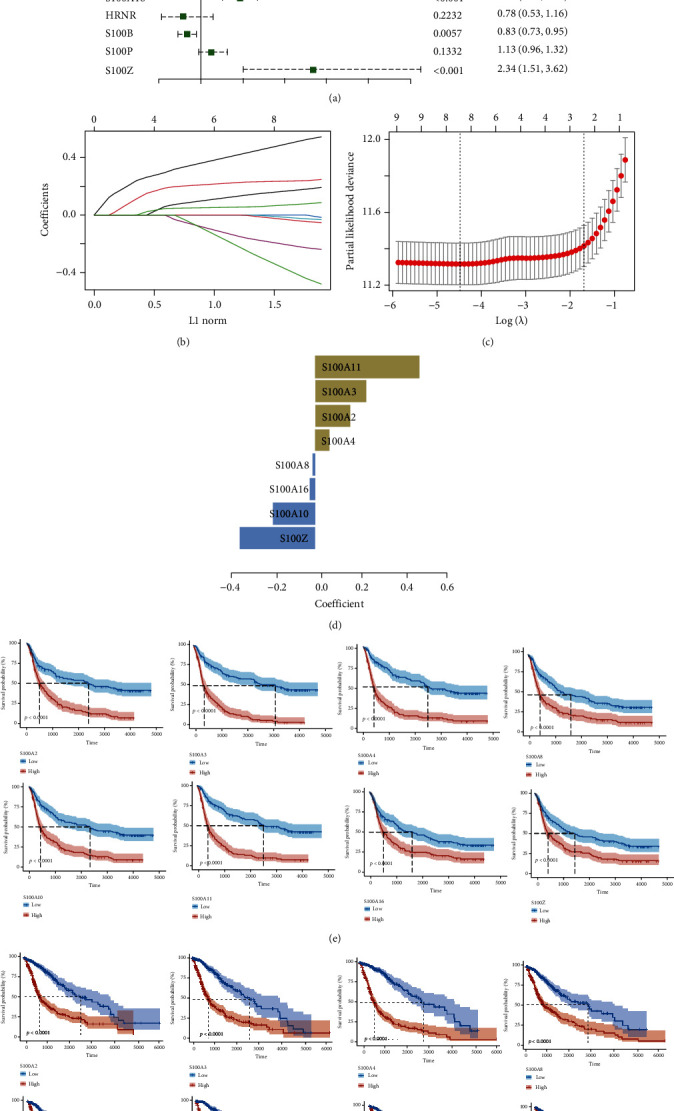
Identification of S100 family genes related to overall survival of glioma. (a) In the CGGA cohort, 17 S100 protein family genes were analyzed by univariate cox analysis. (b) LASSO coefficient profiles of 9 S100 family genes. (c) Partial likelihood deviance of different variables revealed by the LASSO regression model. (d) Bar plot displaying the coefficients constructed using the LASSO method. K-M OS curves of 8 S100 family genes were drawn in the (e) CGGA cohort and (f) TCGA cohort, respectively.

The S100 protein family genes that were significantly correlated with patient's prognosis were determined by performing univariate Cox regression analysis in the CGGA dataset. Nine S100 family genes (*P* < 0.001) were significantly related to prognosis and identified as risk factors for glioma (HR > 1) (Figure 3(a)). The LASSO Cox regression algorithm was subsequently used to analyze nine prognosis-related S100 protein family genes, and finally eight genes were screened out based on the minimum criteria (Figures 3(b)–3(d)). In addition, Kaplan-Meier (K-M) analysis confirmed that all eight S100 protein family genes were significantly related to patients' OS in the CGGA and TCGA datasets (Figures 3(e) and 3(f)).

**Figure 4:**
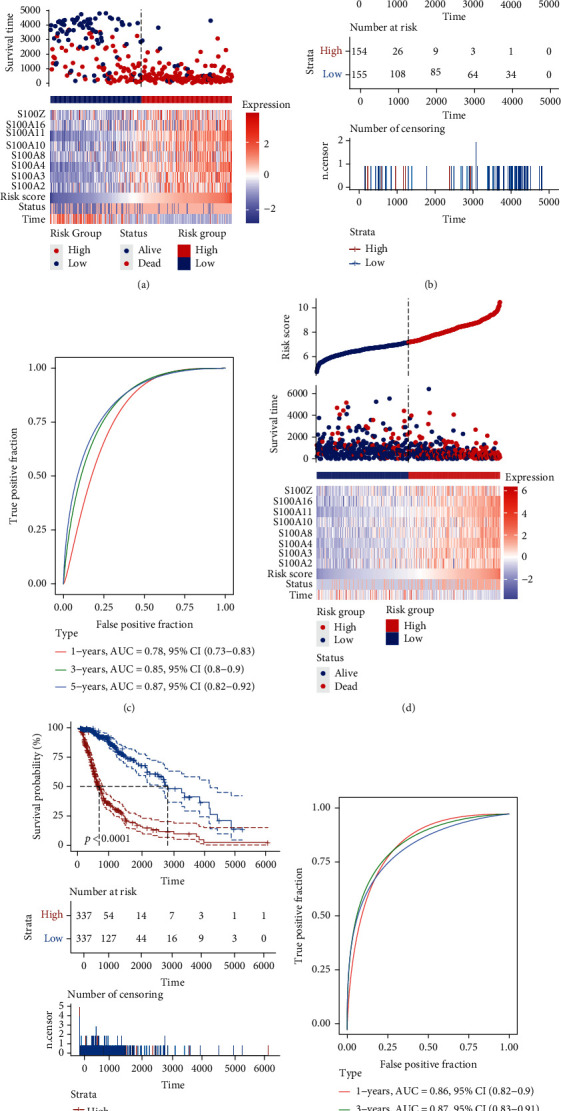
Construction of the risk-score signature using 8 S100 family genes. (a) The expression of 8 signature S100 family genes, survival status, and risk score of each patient in the CGGA cohort. (b) K-M OS curves of different risk subgroups in the CGGA cohort. (c) ROC curves showing the sensitivity and specificity of risk score in predicting the OS of glioma patients at 1-, 3- and 5-year in CGGA cohort. (d) The expression of 8 signature S100 family genes, survival status, and risk score of each patient in the TCGA cohort. (e) K-M analysis of different risk subgroups in the TCGA cohort. (f) ROC curves showing the sensitivity and specificity of risk score in predicting the OS of glioma patients at 1-, 3-, and 5-year in TCGA cohort.

The risk-score signature was constructed according to the eight S100 protein genes and coefficients screened by LASSO (Figure 4(a)). Additionally, the risk model was validated in the TCGA GBMLGG and CGGA #693 cohorts (Figure 4(d) and Supplementary Figure 1A). According to the median risk score, glioma patients were divided into the low-risk subgroup and high-risk subgroup. Kaplan-Meier analysis was then performed to determine the difference in OS between different risk subgroups. The glioma patients' OS in high-risk subgroup was worse and much shorter than that in the low-risk subgroup (Figures 4(b) and 4(e), and Supplementary Figure 1C). Thereafter, we assessed the sensitivity and specificity of risk score in prediction of the overall survival of glioma patients at 1-, 3-, and 5-year. The ROC curves indicated that the risk-score signature was accurate in the prediction of glioma patients' OS in the CGGA cohort and TCGA cohort (Figures 4(c) and 4(f), and Supplementary Figure 1B).

**Figure 5:**
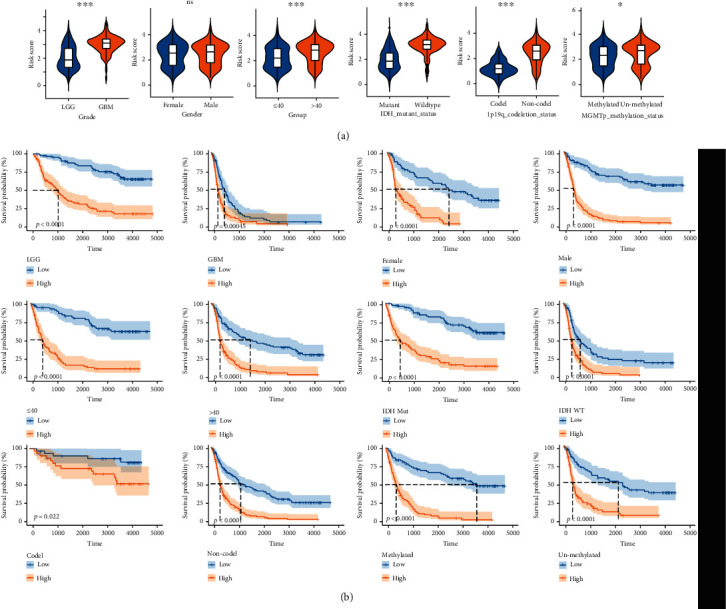
The prognostic analysis of the risk score in different subtypes. (a) Analysis of the risk scores in different IDH status, grade, gender, age, MGMT status, and 1p/19q status subtypes. (b) K-M OS curves of different risk subgroups in different subtypes compartmentalized by grade, gender, age, IDH status, 1p/19q status, and MGMT status. ∗*P* < 0.05; ∗∗*P* < 0.01; ∗∗∗*P* < 0.001.

The clinicopathological characteristics of glioma correlate with prognosis, so we analyzed the risk scores of gliomas in different subtypes compartmentalized by different grade, gender, age, MGMT status, 1p/19q status, and IDH status. The risk scores of gliomas in GBM, age > 40, IDH wild-type, and 1p/19q noncodel and MGMT promoter unmethylated subtypes were significantly higher than those of the corresponding subtypes (Figure 5(a)). In addition, the risk-score signature also exhibited high prognostic value in different separated subtypes (Figure 5(b)).

### 3.3. Immune Landscape of Glioma

**Figure 6:**
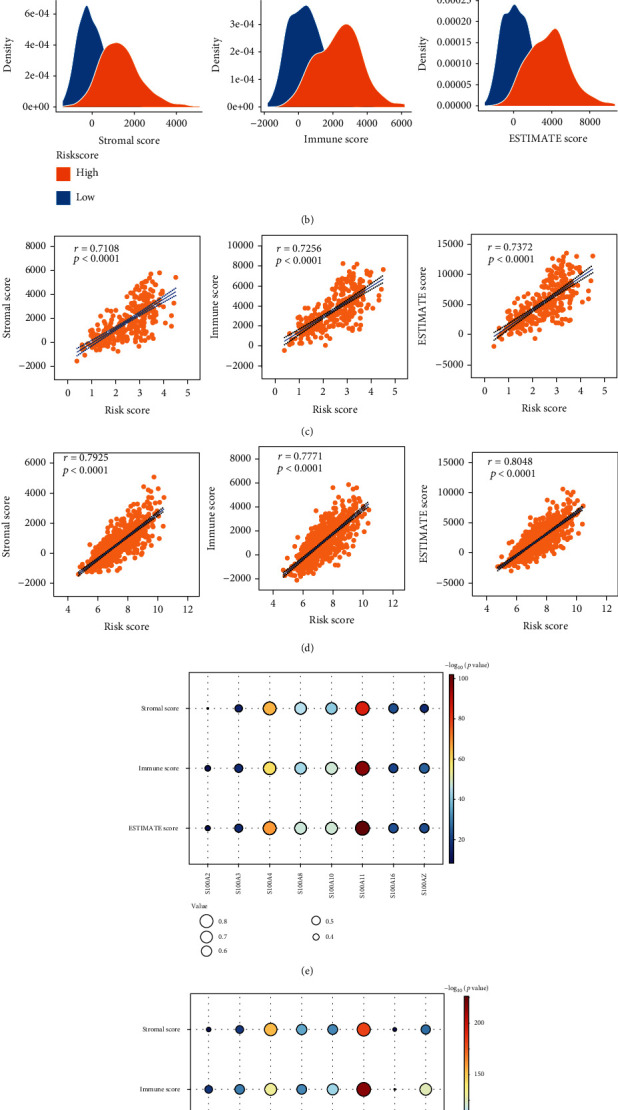
Immune and stromal signatures of gliomas. Ridge plot showing the immune, ESTIMATE, and stromal scores of different risk-score subgroups in the (a) CGGA cohort and (b) TCGA cohort, respectively. Correlation analysis of risk score with immune, ESTIMATE, and stromal scores in the (c) CGGA cohort and (d) TCGA cohort, respectively. Matrix bubble displaying the correlation analysis of the eight S100 family genes with the infiltration levels of T cell CD4+ naïve, Tregs, NK activated, monocyte, neutrophil, and macrophages M0/M1/M2 in (e) the CGGA cohort and (f) the TCGA cohort. ∗*P* < 0.05; ∗∗*P* < 0.01; ∗∗∗*P* < 0.001; ∗∗∗∗*P* < 0.0001.

**Figure 7:**
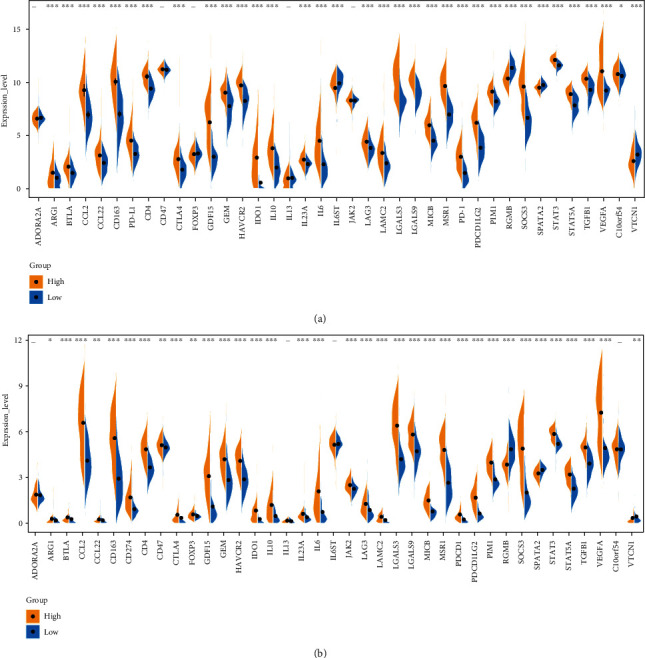
The expression profiles of the immune signature genes in glioma. Violin illustration displaying the expression profiles of 39 immune signature genes between different risk subgroups in the (a) CGGA cohort and (b) TCGA cohort, respectively. ∗*P* < 0.05; ∗∗*P* < 0.01; ∗∗∗*P* < 0.001.

S100 protein family participates in multiple pathological and physiological processes, such as immunity and inflammatory response. Therefore, the risk-score signature might be correlated with the TME in glioma. To test the hypothesis, we analyzed the distribution of stromal, immune, and ESTIMATE scores of glioma patients in different subgroups. Compared with patients in the low-risk subgroup, stromal, immune, and ESTIMATE scores of the high-risk subgroup were significantly increased (Figures 6(a) and 6(b)). Correlation analysis suggested that the risk scores were significantly associated with the stromal scores, immune scores, and ESTIMATE scores in the CGGA and TCGA datasets (Figures 6(c) and 6(d)). In addition, the expression levels of the eight prognostic S100 protein family genes were also significantly correlated with the stromal scores, immune scores, and ESTIMATE scores (Figures 6(e) and 6(f)). To characterize whether the risk scores were associated with the suppressive immunophenotype, we explored the expression of 39 immunosuppressive genes in the CGGA and TCGA datasets. Almost all immunosuppressive genes were upregulated in the high-risk subgroup, including the checkpoint genes PDCD1 (PD-1), CTLA-4, and CD274 (PD-L1) (Figures 7(a) and 7(b)), which suggested the correlation between S100 protein-related signature and immunosuppressive microenvironment.

**Figure 8:**
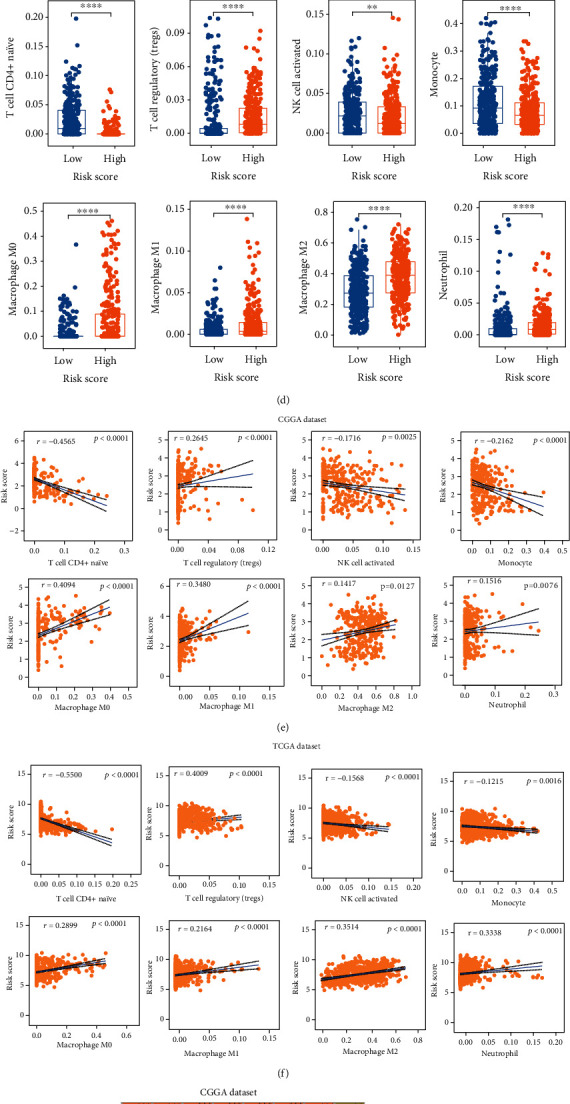
Analysis of 22 infiltrating immune cells in different subgroups divided according to risk score. (a, b) The average frequencies of 22 immune cells of glioma patients in different subgroups were analyzed by TIMER2. Correlation analysis of 8 S100 family genes with the infiltrating immune cells in the (c) CGGA cohort and (d) TCGA cohort, respectively. Correlation analysis of the risk scores with 8 infiltrating immune cells in (e) the CGGA cohort and (f) the TCGA cohort, respectively. Boxplot displaying the infiltration levels of T cell CD4+ naïve, macrophages M0/M/M2, Tregs, monocyte, and neutrophil and NK activated between the different risk subgroups in the (g) CGGA cohort and (h) TCGA cohort, respectively. ∗*P* < 0.05; ∗∗*P* < 0.01; ∗∗∗*P* < 0.001; ∗∗∗∗*P* < 0.0001.

Thereafter, we analyzed twenty-two types of immune cells in gliomas using the CIBERSORT algorithm in the online tool TIMER2 (Figure 8). We found that tumor-infiltrating leukocytes, including macrophages M0/M1/M2, T cell CD4+ naïve, Tregs, monocyte, neutrophil, and NK activated, differed significantly between the different risk subgroups. In detail, Tregs, macrophages M0/M1/M2, and neutrophil infiltration were increased in the high-risk subgroup, while T cell CD4+ naïve, monocyte, and NK activated were decreased (Figures 8(a)–8(d)). Through correlation analysis, we found that the infiltration levels of these eight types of immune cells were significantly associated with risk scores (Figures 8(e) and 8(f)). Moreover, the immune cell infiltration was also significant related to the expression of eight prognostic S100 protein family genes (Figures 8(g) and 8(h)).

### 3.4. S100-Related Signature Is an Independent Risk Factor for Glioma

**Figure 9:**
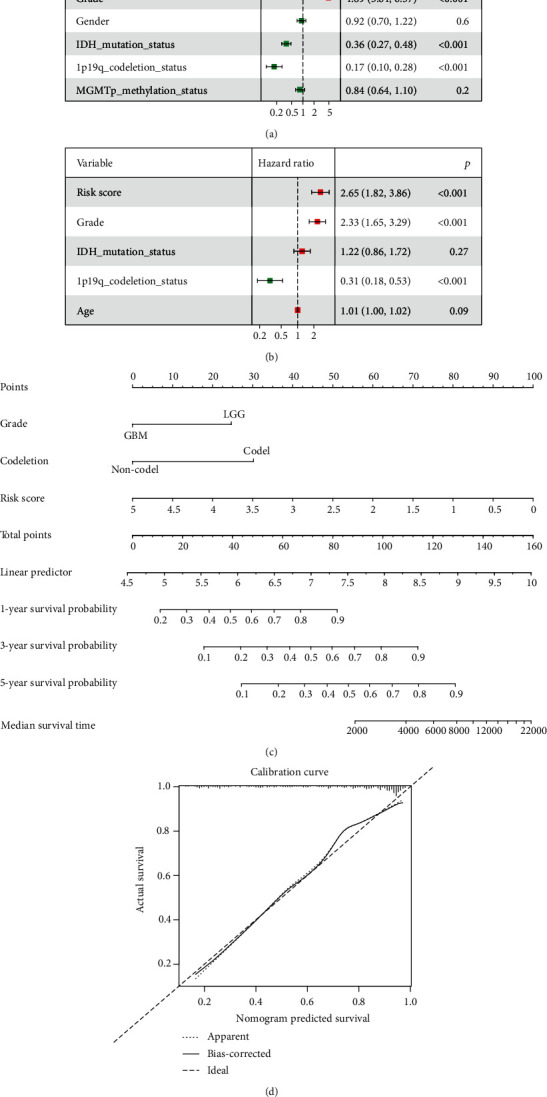
Risk score is an independent prognostic factor for glioma. (a) The clinical features in the CGGA cohort were analyzed by univariate cox regression analysis. (b) The positive clinical features and risk signature in the CGGA cohort were then analyzed by multivariate cox analysis. (c) The nomogram was used to predict the prognosis of patients in the CGGA cohort at 1-, 3-, and 5-year. (d) The calibration curve was drawn to displaying the effect of nomogram in predicting the OS of glioma patients in the CGGA cohort.

We performed univariate Cox regression analysis to investigate the independent prognostic factors for glioma. The results showed that grade, risk score, age, IDH status, and 1p/19q status were significantly correlated with OS of glioma patients (Figure 9(a)). In addition, multivariate analysis showed that the risk score, grade, and 1p/19q status were still closely related to OS (Figure 9(b)). A survival nomogram prediction model was established based on these positively independent prognostic parameters of glioma in CGGA cohort (Figure 9(c)), and the calibration curve displayed excellent agreement between observation and prediction (Figure 9(d)). Taken together, these results suggested the risk-score signature was a reliable prognostic marker for gliomas.

### 3.5. Verify the Expression of the Prognostic S100 Genes

**Figure 10:**
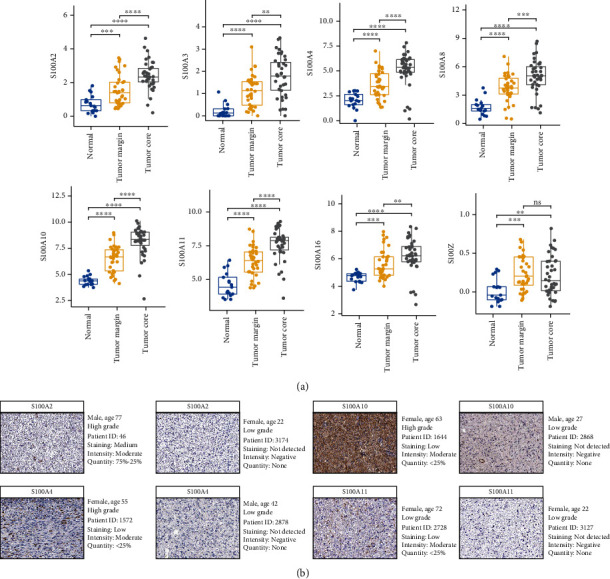
Validation the expression profiles of the prognostic S100 protein family genes. (a) Boxplot showing the expression profiles of eight prognostic S100 family genes between paracancerous tissue, tumor marginal tissues, and tumor core tissue in the GSE59612 dataset. (b) Immunohistochemical staining analysis of the protein levels of S100A2, S100A4, S100A10, and S100A11 between LGG and GBM. ∗*P* < 0.05; ∗∗*P* < 0.01; ∗∗∗*P* < 0.001; ∗∗∗∗*P* < 0.0001.

We analyzed the expression of prognostic S100s in paracancerous tissues, tumor marginal tissues, and tumor core tissues and found that the expression levels of S100A2-4, S100A8, S100A10-11, and S100A16 were significantly increased from paracancerous tissue, tumor marginal tissues to tumor core tissues (Figure 10(a)). Although the expression of S100Z was not significantly different between tumor marginal tissues and tumor core tissues, the expression level was significantly increased in tumor core tissues and tumor marginal tissues relative to paracancerous tissues (Figure 10(a)). To further validate the expression levels of prognostic S100 protein family genes in glioma, we downloaded and analyzed the immunohistochemistry pathological specimen data from the Human Protein Atlas. The results showed that the expression levels of S100A2, S100A4, and S100A10-11 were increased in GBM relative to LGG (Figure 10(b)).

### 3.6. S100A4 Affects Migration and Proliferation of Glioma Cells

**Figure 11:**
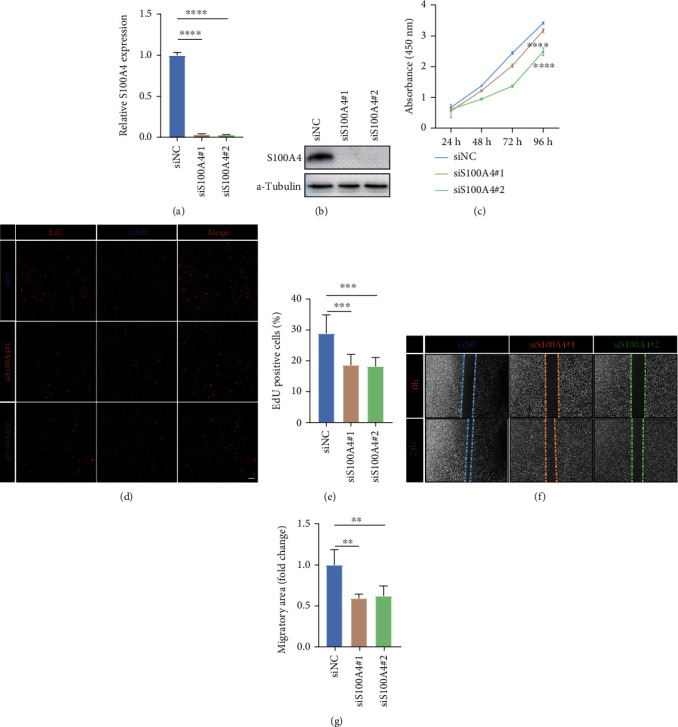
S100A4 affects proliferation and migration of glioma cells. (a) qRT-PCR and (b) western blot analysis of S100A4 knockdown efficiency in LN229 cells. (c) Analysis of proliferation of control and S100A4-deficient LN229 cells by CCK8 assay. (d) Representative images and (e) statistical analysis of EdU assay in control and S100A4-deficient LN229 cells. (f) Representative images and (g) statistical analysis of cell migration assay in control and S100A4-deficient endothelial cells at the indicated times. ∗*P* < 0.05; ∗∗*P* < 0.01; ∗∗∗*P* < 0.001; ∗∗∗∗*P* < 0.0001.

To fully determine the effect of S100A4 on glioma cells, we knocked down S100A4 in LN229 cells by transfecting specific siRNA (Figures 11(a) and 11(b)). CCK8 and EdU assays showed that S100A4 could significantly affect the proliferation of LN229 cells (Figure 11(c)–11(e)). In addition, knockdown S100A4 significantly inhibited the migration of LN229 cells (Figures 11(f) and 11(g)).

## 4. Discussion

Gliomas are the most common malignant central nervous system tumors. Conventional therapies such as surgery, chemotherapy, and radiotherapy cannot effectively improve prognosis of glioma patients [22]. Chimeric antigen receptor T-cell immunotherapy (CAR-T) is considered a great potential therapeutic method [23]. Moreover, immune checkpoint inhibitors such as anti-PD1 antibody, anti-PDL1 antibody, and anti-CTLA4 antibody have made great progress in the clinical treatment of some solid tumors [24–26]. However, highly immunosuppressive TME and immune evasion are still the huge challenges for immunotherapy of glioma patients. Therefore, additional research is needed to uncover the mechanism of suppressive TME in glioma and find new prognostic biomarkers and therapeutic strategies.

S100 protein family participates in multiple pathological and physiological processes, including apoptosis, inflammatory reaction, and cancer progression [27]. Previous studies have reported the potential prognostic role of the S100A genes and the S100 family genes [28, 29]. In current study, we constructed a novel prognostic model based on eight S100 family genes (S100A2-4, S100A8, S100A10-11, S100A16, and S100Z) and demonstrated its effectiveness in predicting the prognosis of gliomas (Figure 4 and Supplementary Figure 1).

S100A4 is involved in the process of chemokine and cytokine-like activities after being secreted to the extracellular space [30, 31]. The serum level of S100A8/A9 complex is significantly elevated during wound healing process, tumorigenesis, and autoimmune diseases [32] and is used as an extremely sensitive biomarker for the early stage of local inflammatory activity [33]. The released extracellular S100A8/A9 stimulates monocytes and macrophages, leading to increased production of proinflammatory cytokines [34]. S100A11 induces chemokine response and regulates monocyte recruitment in vivo [35]. Therefore, S100 family proteins may be involved in increased inflammatory cell infiltration and remodeling of the TME in gliomas.

We analyzed the relationship between the risk signature and the landscape of immune microenvironment in gliomas and found that the risk scores were significantly positively correlated with the immune scores, ESTIMATE scores, and stromal scores (Figure 6). Almost all immunosuppressive genes, including the checkpoint genes such as PD1, PDL1, and CTLA4, were upregulated in the high-risk subgroup (Figure 7), indicating that the risk signature was related to the suppressive immunophenotype. In addition, the high-risk subgroup had increased levels of Tregs, macrophages M0/M1/M2, and neutrophil infiltration and decreased T cell CD4+ naïve, NK activated, and monocyte infiltration (Figure 8), reflecting the local immunosuppressive microenvironment of gliomas. Dysregulation of S100 family proteins may contribute to the inflammatory status, while chronic inflammation promotes tumor progression, metastasis, and drug resistance by reorganizing the tumor immune microenvironment [36]. In summary, our findings provide a novel insight into the relationship between S100 family proteins and immunosuppressive microenvironment and provide potential targets for treatment of gliomas.

## Data Availability

The public data analyzed in our research could be found on the following websites: (https://www.ncbi.nlm.nih.gov/geo/;http://www.cgga.org.cn/;https://xenabrowser.net/datapages/).

## Supplementary Materials

Supplementary Materials Supplementary Figure 1: validation of the risk-score signature on CGGA #693 cohort. (A) The expression of 8 signature S100 family genes, survival status, and risk score of each patient in the CGGA #693 cohort. (B) ROC curves showing the sensitivity and specificity of risk score in predicting the OS of glioma patients at 1-, 3- and 5-year in CGGA #693 cohort. (C) K-M curves of different risk subgroups in the CGGA #693 cohort. Supplementary Table 1: siRNA used in this study.
