# Supplementary figures and images for: Alarm Signal S100-Related Signature Is Correlated with Tumor Microenvironment and Predicts Prognosis in Glioma
Source: Dis Markers. 2022 May 10;2022:4968555. doi: 10.1155/2022/4968555 (PMC9113871; doi:10.1155/2022/4968555)

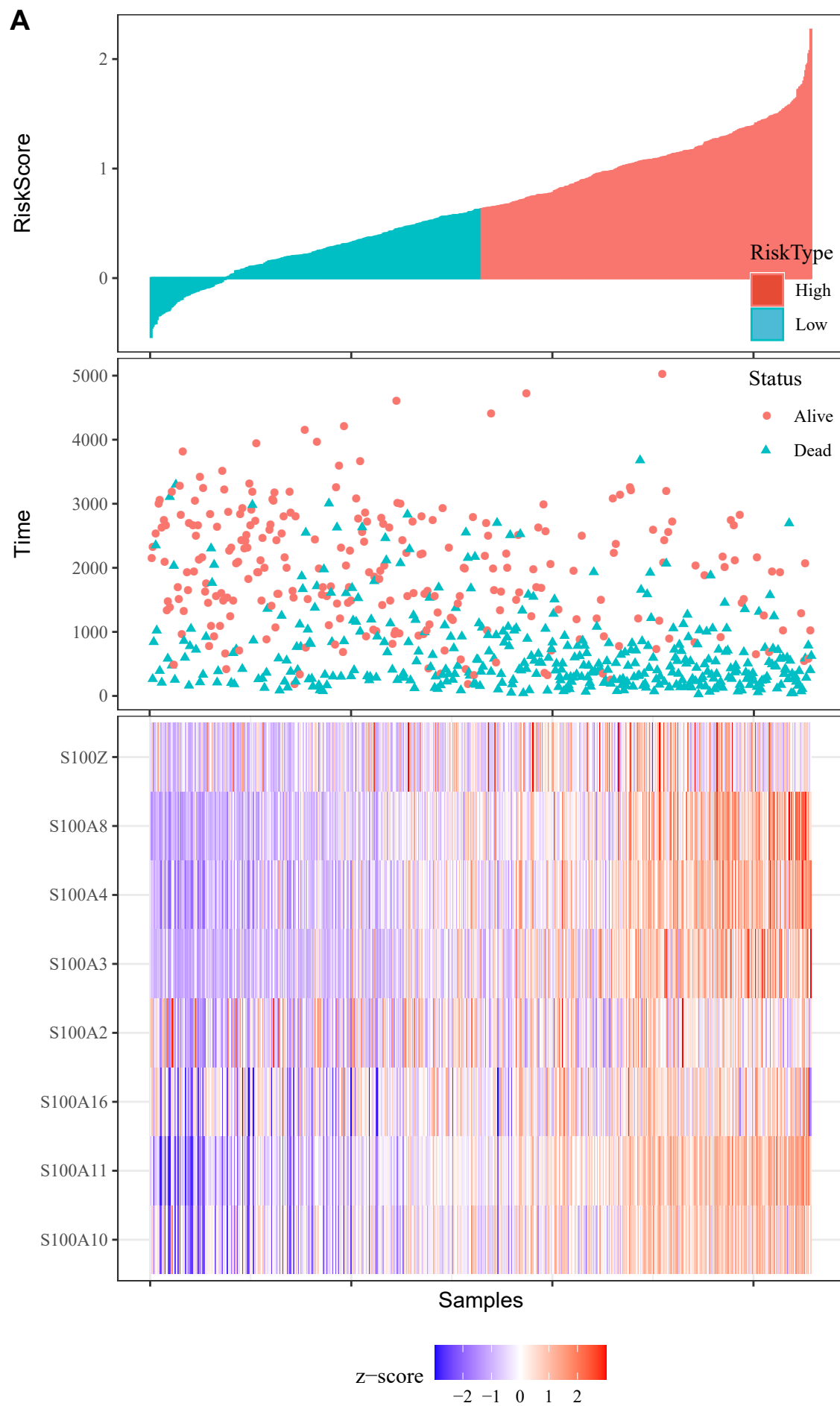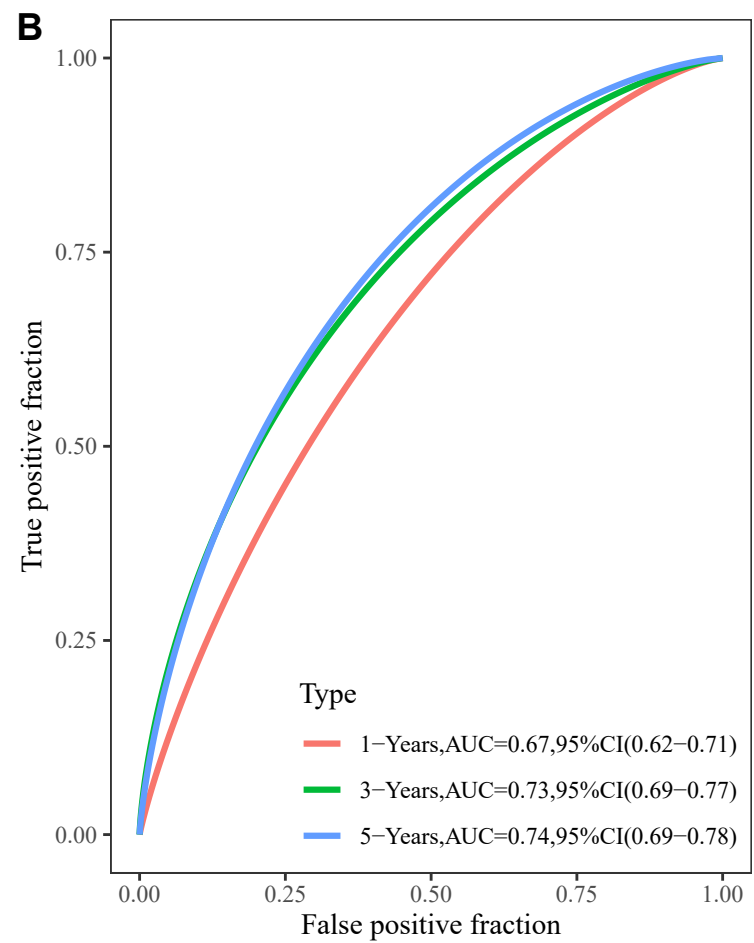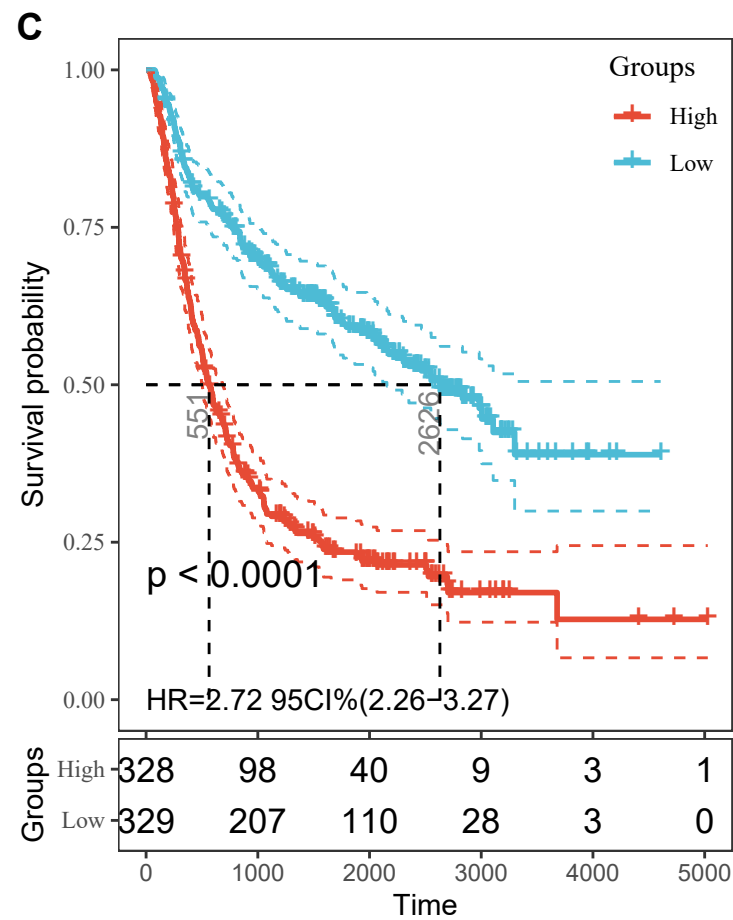

Supplement: Supplementary Materials — Supplementary Figure 1: validation of the risk-score signature on CGGA #693 cohort. (A) The expression of 8 signature S100 family genes, survival status, and risk score of each patient in the CGGA #693 cohort. (B) ROC curves showing the sensitivity and specificity of risk score in predicting the OS of glioma patients at 1-, 3- and 5-year in CGGA #693 cohort. (C) K-M curves of different risk subgroups in the CGGA #693 cohort. Supplementary Table 1: siRNA used in this study. [file 4968555.f1.pdf]
